# Impact of immunosuppression on the incidence of ventilator-associated events: an observational study

**DOI:** 10.1186/s12871-026-03804-0

**Published:** 2026-04-07

**Authors:** Sergei Vladimirov, Ilia Klimenko, Nikita Matiushkov, Denis Protsenko, Dmitry Sergeev

**Affiliations:** 1 Saint Petersburg Information Technologies, Mechanics and Optics University (ITMO) University, Kronverkskiy prospekt, 49, St. Petersburg, 197101 Russia; 2 Kommunarka Moscow multi-purpose clinical center, Sosensky Stan Street, bldg 8, Moscow, 108814 Russia; 3 Pirogov Russian National Research Medical University, Ostrovityanova ulitsa,1 bldg. 6, Moscow, 117513 Russia; 4 Personalized Early Detection of Prostate Cancer , German Cancer Research Center (DKFZ), Heidelberg, Germany; 5 Medical Faculty Heidelberg, University of Heidelberg, Heidelberg, Germany

**Keywords:** Ventilator-associated events, Immunosuppression, Mechanical ventilation

## Abstract

**Background:**

Critically ill immunocompromised patients requiring mechanical ventilation (MV) may be particularly vulnerable to ventilator-associated events (VAE), yet the impact of baseline immunosuppression on VAE incidence remains unclear.

**Methods:**

We conducted a secondary analysis of a cohort of adults requiring MV for ≥4 days in two ICUs of a tertiary hospital in Moscow, Russia. Using competing-risk regression, we compared the 30-day incidence of VAE between immunocompromised and non-immunocompromised patients. We then evaluated the association between VAE and 30-day ICU mortality among immunocompromised patients using Cox regression.

**Results:**

Of 269 patients, 122 (45.4%) had baseline immunosuppression. The incidence of any VAE was higher in immunocompromised patients, though the estimate was imprecise (adjusted subhazard ratio [aSHR] 1.64; 95% CI 0.82–3.30); for infection-related ventilator-associated complications (IVAC), the estimate suggested a more than twofold increased hazard, but with considerable uncertainty (aSHR 2.22; 95% CI 0.85–5.78). In patients with immunosuppression, IVAC were associated with increased mortality (adjusted hazard ratio 2.38; 95% CI 1.16–4.89).

**Conclusion:**

Despite a higher estimated incidence of VAE in immunocompromised patients, we could not establish a clear association between baseline immunosuppression and VAE risk. IVAC were associated with increased mortality in immunocompromised patients, although this finding should be considered exploratory.

**Supplementary Information:**

The online version contains supplementary material available at 10.1186/s12871-026-03804-0.

## Introduction

Immunocompromised patients have experienced improved intensive care unit (ICU) outcomes over the past two decades, yet those requiring mechanical ventilation (MV) still face high mortality [1, 2]. Their underlying immune dysfunction, often combined with greater disease severity and poor baseline status, may predispose them to MV-related complications, including ventilator-associated events (VAE), and adverse outcomes [3, 4].

Previous studies have reported conflicting findings regarding the risk of ventilator-associated lower respiratory tract infections (VA-LRTI) in this population, which introduces uncertainty for everyday ICU practice. An analysis of a multicenter cohort reported lower rates of ventilator-associated tracheobronchitis (VAT) and ventilator-associated pneumonia (VAP), whereas another international study found higher rates of hospital-acquired infections (HAI) but not VA-LRTI among immunocompromised patients [5, 6].

In these patients, diagnostic assessment is further complicated by blunted inflammatory responses and the subjective nature of VAP criteria [7–9]. In addition, several non-infectious conditions can closely mimic pneumonia (such as pulmonary drug toxicity, acute pulmonary edema, leukemic infiltration, and diffuse alveolar hemorrhage), making reliable surveillance of VA-LRTI challenging [10–12].

To address the limitations of traditional VA-LRTI definitions, the Centers for Disease Control and Prevention (CDC)/National Healthcare Safety Network (NHSN) introduced ventilator-associated events (VAE), which have been consistently associated with worse clinical outcomes [9, 13, 14]. Most VAE studies have evaluated mixed ICU populations, few have focused specifically on patients who are already immunocompromised at ICU admission, and little is known about how different VAE tiers influence outcomes in this group.

Therefore, this study aimed to determine whether immunocompromised status at ICU admission is associated with an increased risk of VAE among patients undergoing prolonged MV.

## Methods

### Study design and population

We have previously published a cohort study investigating the incidence and clinical outcomes of VAE in adults receiving prolonged MV [15]. That study was conducted at the Kommunarka Moscow Multi-Purpose Clinical Center (Kommunarka MMCC), a high-volume tertiary care hospital in Moscow, Russia, which also serves as a referral center for hematology and oncology services. The original research used electronic health record (EHR) data from two 16-bed ICUs and included patients admitted between September 1, 2022, and December 31, 2023. The current paper presents a secondary analysis of this established cohort.

### Definition of immunosuppression

Immunocompromised status was defined based on ICD-10 diagnoses recorded at ICU admission, including solid organ malignancy, hematologic malignancy, systemic autoimmune disease, HIV infection, or any history of solid organ or stem cell transplantation. The full list of ICD-10 codes is provided in Supplementary Table 1.

### Identification of VAE

Only patients who received at least four consecutive calendar days of MV were eligible for VAE assessment. VAE were identified and classified according to CDC/NHSN surveillance criteria [13] into three hierarchical tiers: ventilator-associated condition (VAC), infection-related ventilator-associated complication (IVAC), and possible ventilator-associated pneumonia (PVAP). IVAC-plus included events meeting criteria for IVAC or PVAP. Candidate events were first identified using a custom R script to detect CDC/NHSN-defined changes in positive end-expiratory pressure (PEEP) and fraction of inspired oxygen (FiO₂). Two authors then independently reviewed the corresponding charts and applied CDC/NHSN criteria to confirm VAC and classify IVAC and PVAP; disagreements were resolved by consensus with a third investigator [15].

### Study outcomes

The primary outcome was the cumulative incidence of any VAE (a composite of VAC, IVAC, and PVAP – total VAE, or VAE-plus) by day 30 after initiation of MV, compared between immunocompromised and non-immunocompromised patients. Patients were followed from the start of MV until ICU discharge or day 30, whichever occurred first.

Secondary outcomes included:

- The cumulative incidence of each individual VAE tier, compared between the two groups.
- The impact of developing VAE on 30-day mortality, ICU length of stay (LOS), and MV duration from MV initiation among immunocompromised patients.
- The spectrum of new antimicrobial agents started at IVAC-plus events and the distribution of microorganisms isolated in PVAP episodes, extracted from electronic medication orders and microbiology reports.

VAE incidence was assessed during the first MV episode per ICU admission. An MV episode was considered continuous if interruptions lasted less than 2 calendar days; consequently, successful extubation was defined as liberation from MV for at least this same 2-day period. Patients who were transferred out of the ICU while still receiving MV and were lost to follow-up were censored on the last day of known MV status. The total duration of MV was calculated as all ventilator days within the 30-day follow-up period.

### Statistical analysis

Baseline characteristics at ICU admission were summarized and compared between groups. Categorical variables are reported as counts and frequencies, and continuous variables as medians with interquartile ranges (IQR). The original cohort study excluded patients with incomplete EHR data; consequently, the present secondary analysis was based on complete cases. Missing data occurred in 17 patients (approximately 6%) due to temporary technical failures; therefore, a complete-case analysis was performed.

We estimated the cumulative incidence of VAE using competing risks methods, treating death and successful weaning from MV as competing events because both preclude subsequent VAE, and treating higher-tier events (e.g., IVAC, PVAP) as competing events when analysing lower-tier outcomes (e.g., VAC). Comparisons between immunocompromised and non-immunocompromised patients were made using Fine-Gray subdistribution hazard models with time on MV as the time scale, with follow-up censored at day 30 if no event occurred. Models were adjusted for the sum of non-immunosuppressive components of the Charlson Comorbidity Index (CCI); no additional covariates were included in the primary analysis because variables reflecting disease severity or the reasons for tracheal intubation may lie on the causal pathway between immunosuppression and VAE and could introduce overadjustment bias. Adjusted subdistribution hazard ratios (aSHR) with 95% confidence intervals (CI) were reported. To assess the robustness of our findings, we performed a sensitivity analysis additionally adjusting for indication for MV and number of days hospitalized before intubation – factors that may confound the relationship between immunosuppression and VAE. Unadjusted estimates were also included as part of this analysis.

To study the effect of VAE on mortality in immunocompromised patients, a Cox regression model with VAE status as a time-dependent covariate was used, accounting for the fact that VAE can occur at different times during MV. Models were adjusted for baseline Sequential Organ Failure Assessment (SOFA) score and CCI, and adjusted hazard ratios (aHR) with 95% CI were reported; this analysis was repeated for each VAE tier.

We compared ICU length of stay and duration of mechanical ventilation using the Wilcoxon test.

A two-sided p-value < 0.05 was considered statistically significant. Analyses were conducted in R (version 4.5.1; R Foundation for Statistical Computing) [16].

### Microbiology data

Respiratory specimens for PVAP assessment included bronchoalveolar lavage (BAL) and endotracheal aspirate (ETA). Samples were collected during the VAE window – on or after calendar day 3 of MV and within 2 calendar days before or after the onset of worsening oxygenation. Quantitative cultures were performed, with diagnostic thresholds set at ≥ 10⁴ colony-forming units (CFU])/ml for BAL and ≥ 10⁵ CFU/ml for ETA, in accordance with CDC/NHSN criteria [15]. Polymicrobial cultures were considered positive only if all isolated organisms met the quantitative thresholds. Organisms not meeting PVAP criteria (e.g., Candida species, coagulase-negative staphylococci, Enterococcus species) were excluded, as specified by CDC guidelines.

Species identification of isolated microorganisms was performed by matrix-assisted laser desorption/ionization time-of-flight mass spectrometry (MALDI-TOF MS). Antimicrobial susceptibility testing was conducted using the disk diffusion method, and results were interpreted according to European Committee on Antimicrobial Susceptibility Testing (EUCAST) [17] criteria valid at the time of the study. Multidrug-resistant (MDR), extensively drug-resistant (XDR), and pandrug-resistant (PDR) phenotypes were defined according to the international consensus criteria [18].

### Ethical considerations

The original study protocol was approved by the Local Ethics Committee of Kommunarka MMCC (Protocol №6, dated September 24, 2024). The requirement for informed consent was waived due to the retrospective, anonymized nature of the data collection. For this secondary analysis of fully de-identified data, separate ethical approval was not required.

## Results

### Study population

**Fig. 1:**
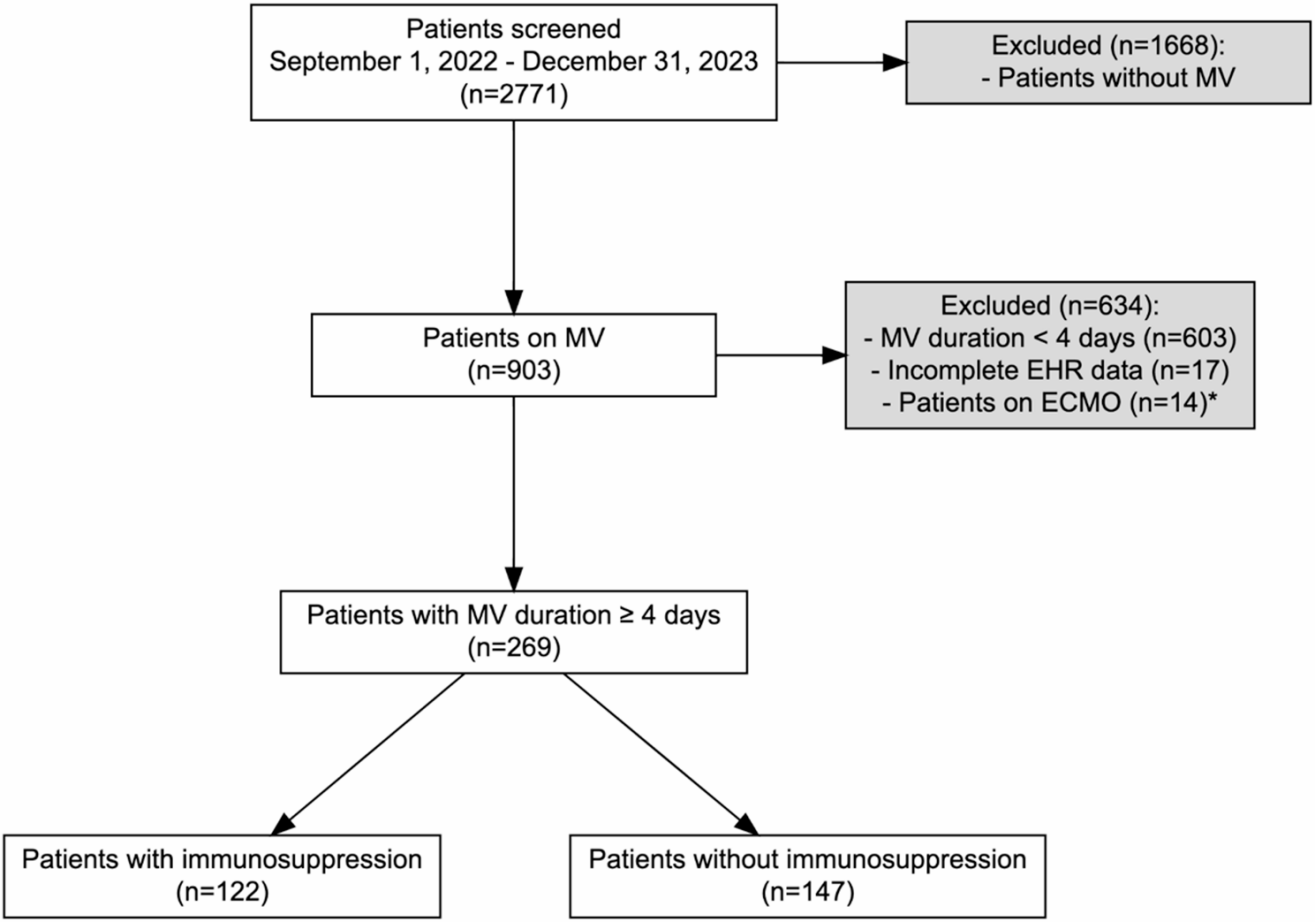
Study flowchart. Abbreviations: MV, mechanical ventilation; EHR, electronic health record; ECMO, extracorporeal membrane oxygenation. *ECMO patients were excluded because they did not contribute any calendar days with conventional MV eligible for VAE surveillance, in accordance with CDC/NHSN criteria

A total of 269 patients received at least four consecutive days of MV, of whom 122 (45.4%) were classified as immunocompromised (Fig. 1). Demographic characteristics and illness severity at ICU admission were similar between groups, with slightly more than half of patients being male and a median SOFA score of 7.0. However, immunocompromised patients had a higher comorbidity burden (median CCI 9 vs. 5) and a longer hospital stay before intubation (median 5 vs. 1 days). Median ICU LOS after initiation of MV was 14 (8–26) days. Respiratory failure was the most frequent indication for MV among immunocompromised patients, whereas neurological deterioration predominated in the non-immunocompromised group; detailed characteristics are presented in Table 1. Solid organ and hematologic malignancies were the most common immunocompromised conditions; the ICD-10 codes used to define baseline immunosuppression are listed in Supplementary Table 1.

**Table 1:** Baseline characteristics of patients included in the study

Characteristic	Non-immunocompromised (*n* = 147)	Immunocompromised (*n* = 122)
Demographics		
Age, mean (SD), years	62 (19)	66 (13)
Male sex, n (%)	80 (54%)	67 (55%)
Clinical status at ICU admission		
SOFA score, median (IQR)	7 (4–9)	7 (4–10)
CCI score, median (IQR)	5 (2–8)	9 (6–12)
Admission characteristics		
Post-operative admission, n (%)	72 (49%)	56 (46%)
Days in hospital before MV initiation, median (IQR)	1 (1–6)	5 (2–14)
Indication for MV, n (%)		
Respiratory failure	43 (29%)	70 (57%)
Neurological deterioration	65 (44%)	19 (16%)
General anesthesia	28 (19%)	24 (20%)
Cardiac arrest	11 (8%)	9 (7%)
ICU outcomes by day 30 from the MV start		
Dearth, n (%)	71 (48.3%)	92 (75.4%)
MV duration in days, median (IQR)	12 (6.0–22.5)	10 (6.0–21.5)
ICU LOS in days, median (IQR)	15 (9.0–27.5)	13 (7.0–23.0)

*Abbreviations*: *CCI* Charlson Comorbidity Index, *CPR* cardiopulmonary resuscitation, *ICU* intensive care unit, *IQR* interquartile range, *LOS* length of stay, *MV* mechanical ventilation, *SD* standard deviation, *SOFA* Sequential Organ Failure Assessment

### ​Ventilator-associated events

**Fig. 2:**
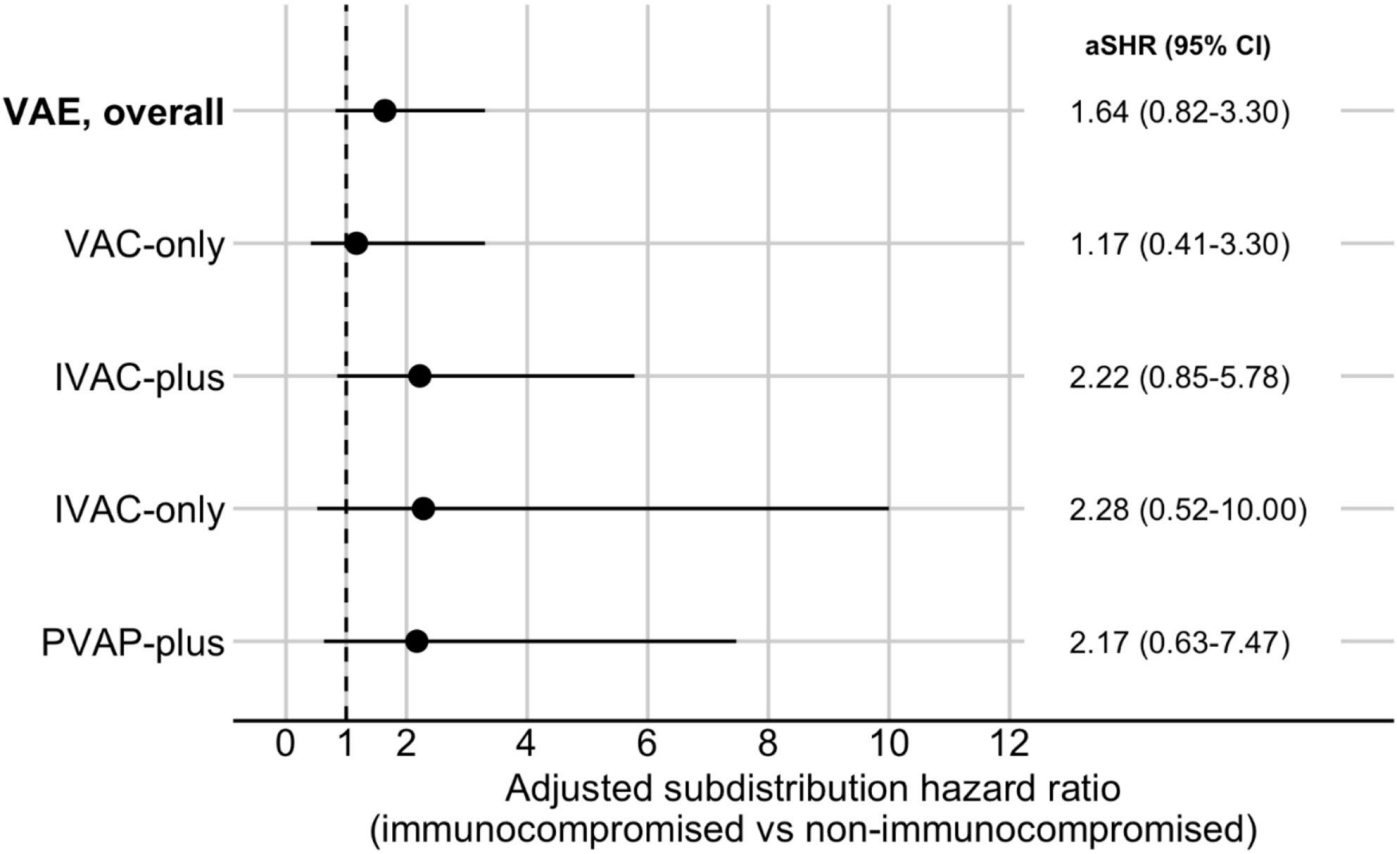
Association between immunocompromised status and ventilator-associated event incidence. Abbreviations: CI, confidence interval; aSHR, adjusted subdistribution hazard ratio; IVAC, infection-related ventilator-associated complication; PVAP, possible ventilator-associated pneumonia; VAC, ventilator-associated condition; VAE, ventilator-associated event. Categories: Overall VAE, VAE of all tiers; VAC-only, events with VAC criteria and without criteria of subsequent tiers; IVAC-plus, events with IVAC criteria (including patients with PVAP); IVAC-only, patients with IVAC and without PVAP criteria; PVAP-plus, events with PVAP criteria

A total of 35 VAE occurred. The estimated incidence of any VAE was higher in immunocompromised patients (aSHR 1.64; 95% CI 0.82–3.30); however, the confidence interval included the possibility of no difference. When VAE tiers were examined separately, the point estimate for IVAC-plus events suggested more than a twofold increased hazard (aSHR 2.22; 95% CI 0.85–5.78), though this estimate was imprecise. For VAC-only events, the relative risk measure was uncertain (aSHR 1.17; 95% CI 0.41–3.30), as illustrated in Fig. 2.

### Outcomes in immunocompromised patients

Among immunocompromised patients, the mortality point estimate was higher for those who developed any VAE compared with those without VAE, although the confidence interval for this estimate was wide (aHR 1.65; 95% CI 0.94–2.88). Development of IVAC-plus events was associated with higher mortality (aHR 2.38; 95% CI 1.16–4.89); a hazard ratio of similar magnitude was observed for PVAP-plus (aHR 2.25; 95% CI 0.95–5.08), but with less precision. No considerable differences were observed in MV duration or ICU LOS between immunocompromised patients with and without VAE **(**Table 2**)**.

**Table 2:** Outcomes in immunocompromised patients, by day 30 from the start of mechanical ventilation

Outcome	No VAE, n = 103	VAE-plus, n = 19	VAC-only, n = 8	IVAC-plus, n = 11	PVAP-plus, n = 7
Crude ICU mortality, %	74.8	79	62.5	91	86
Adjusted HR^†^ for mortality (95% CI)	Reference	1.65 (0.94–2.88)	1.04 (0.43–2.51)	2.38^*^ (1.16–4.89)	2.25 (0.89–5.69)
MV duration in days, median (IQR)	10 (6–22)	13 (6–18)	10 (6–27)	13 (9–16)	11 (9–14)
ICU LOS in days, median (IQR)	13 (7–24)	13 (9–20)	12.5 (10–27)	13 (9–19)	11 (9–14)

Categories: VAE-plus, patients with VAE; VAC-only, patients with VAC and without criteria of subsequent tiers; IVAC-plus, patients with IVAC criteria (including patients with PVAP); PVAP, patients with PVAP criteria

Abbreviations: CI confidence interval HR hazard ratio, ICU intensive care unit, IQR interquartile range, IVAC infection-related ventilator-associated complication, LOS length of stay, MV mechanical ventilation, PVAP possible ventilator-associated pneumonia, VAC ventilator-associated condition, VAE ventilator-associated event

† Time-dependent Cox proportional hazards model, with VAE status as a time-varying exposure, adjusted for baseline SOFA score and Charlson Comorbidity Index

* Indicates statistical significance (*p*<0.05)

### Antimicrobials and pathogens

**Fig. 3:**
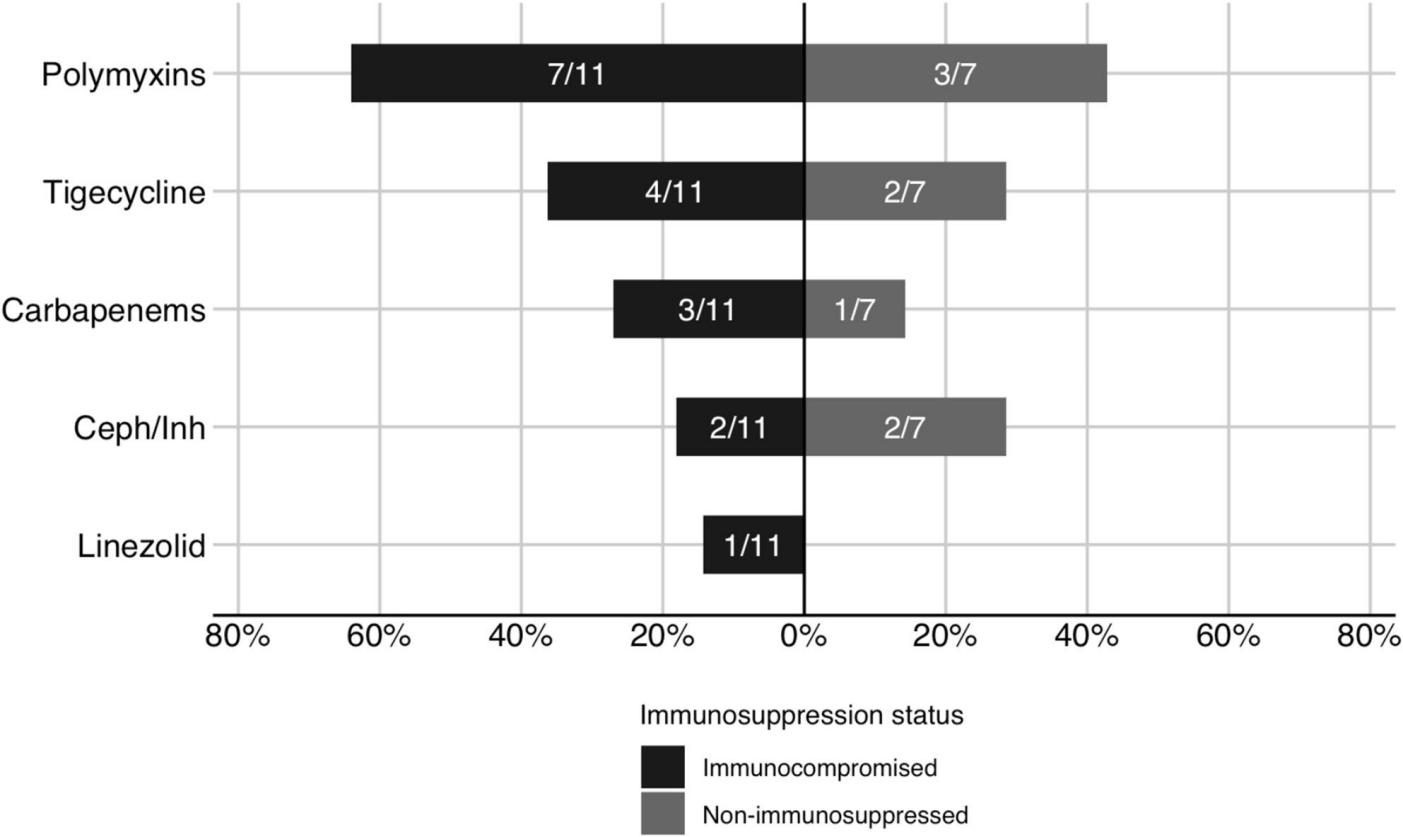
Antimicrobial spectrum of new agents added at infection-related ventilator-associated complication by patient immune status. Numbers within bars indicate the proportion of IVAC-plus episodes treated with each antimicrobial class (treated episodes/total number of IVAC-plus episodes in each group); IVAC-plus, events with IVAC criteria; Ceph/Inn, cephalosporin/β‑lactamase inhibitor combinations

**Fig. 4:**
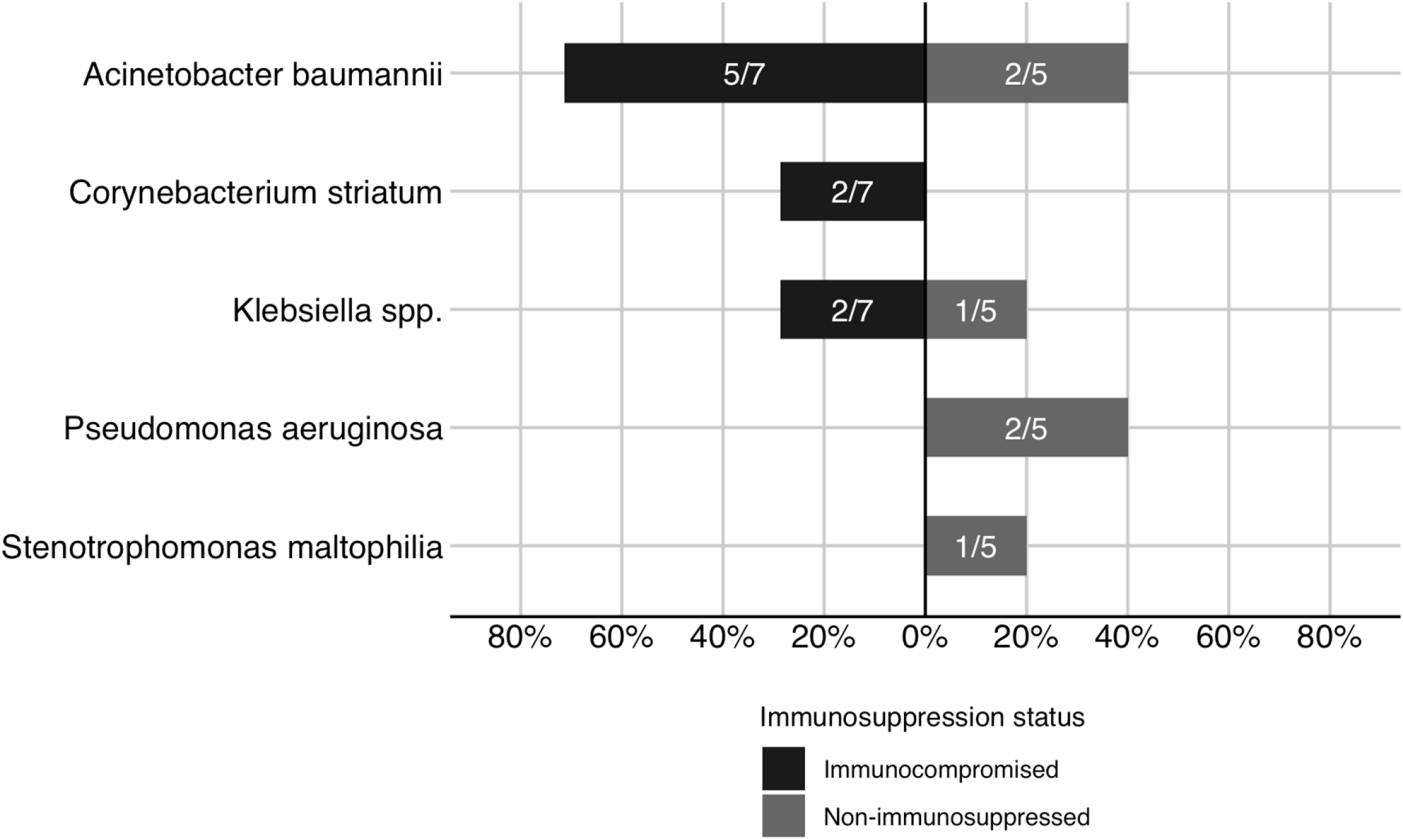
Microbiological etiology of possible ventilator-associated pneumonia by immune status. Numbers within bars show the proportion of possible ventilator‑associated pneumonia episodes associated with each pathogen (pathogen‑specific episodes / total possible ventilator‑associated pneumonia episodes in each group)

Among patients with IVAC, polymyxins were the most frequently used antimicrobials in both immunocompromised and non-immunocompromised groups (Fig. 3). In patients with PVAP, A. baumannii was the most commonly isolated pathogen, with a higher predominance among immunocompromised patients (Fig. 4).

Antimicrobial resistance profiles for key pathogens isolated from PVAP events are presented in Supplementary Tables 2 and 3. All isolates were carbapenem‑resistant and met XDR criteria, with colistin susceptibility retained in all cases.

### Test assumpsions check

The proportional subdistribution hazards assumption in Fine-Gray models and the proportional hazards assumption in Cox models were evaluated using time-varying coefficient plots and formal residual-based tests, with no major deviations from proportionality detected (Supplementary Figs. 1–4, Supplementary Table 4).

### Sensitivity analysis

In sensitivity models additionally adjusting for days hospitalized before intubation and indication for MV, immunosuppression showed an increased hazard of any VAE (aSHR 2.07, 95% CI 1.03–4.17), with directionally similar but imprecise estimates for VAC‑only and IVAC‑plus tiers. Unadjusted estimates were directionally consistent with the primary analysis, but also imprecise (Supplementary Table 5).

## Discussion

In the cohort of 269 mechanically ventilated patients, nearly half met predefined criteria for immunocompromised status. Although the incidence of VAE was higher in immunocompromised patients, the estimates were imprecise and did not allow us to establish a clear association. The largest point estimates were observed for infection-related events (IVAC-plus and PVAP-plus). Among immunocompromised patients, IVAC-plus events suggested an association with higher 30-day ICU mortality; however, this finding should be considered exploratory due to the small number of events.

These results may contribute to the growing literature on VAE risk factors [19–21], within which the role of baseline immunosuppressive conditions has been underexplored. A notable exception is a registry-based cohort study from China [19], which reported that patients with VAE had higher exposure to immunosuppressive drugs than those without VAE, yet found no significant difference in the prevalence of malignancies between patients with and without VAE; however, these data were presented only descriptively and were not evaluated as primary or secondary endpoints. In contrast, our study specifically evaluates immunocompromised status as the primary exposure, using a broad diagnosis-based definition that captures multiple causes of impaired immunity and applying time-to-event methods to quantify its association with VAE incidence.

Our results appear to contrast with several observational studies that have not consistently identified immunosuppression as a risk factor for traditionally defined VA-LRTI [5, 6]. One potential explanation for this discrepancy is that the VAE framework was designed to objectively capture a broader spectrum of nosocomial complications in mechanically ventilated patients, representing a distinct surveillance entity [7]. Its standardized, parameter-based criteria might help mitigate diagnostic challenges inherent in traditional VAP surveillance definitions, which can be particularly misleading in immunocompromised hosts [3]. 

It is important to note, however, that VAE are surveillance constructs and represent a different entity from clinically diagnosed VAP [9]. In particular, IVAC may be triggered by non-pulmonary infections or systemic inflammation rather than reflecting a true pulmonary infection. At the same time, our findings regarding antimicrobials and microbiology align with previous studies on HAI in immunocompromised patients, which indicate a propensity for drug-resistant pathogens. The predominance of polymyxin use in IVAC-plus events and the high frequency of Acinetobacter baumannii in PVAP confirm a substantial burden of MDR/XDR infections in this population [5, 6], reflecting both host vulnerability and ICU-level antimicrobial selection pressure.

While the VAE definition was designed to be polyetiological, we observed a gradient suggesting that the major contribution to VAE incidence in our study was driven by infection-related tiers. Risks for IVAC and PVAP appeared numerically higher in immunocompromised patients, whereas VAC-only events, estimated solely by changes in ventilator parameters, did not differ substantially between groups. This pattern is biologically plausible, given that common mediators in immunocompromised hosts – including neutropenia, qualitative immune dysfunction, prior broad-spectrum antibiotic exposure, and colonization with MDR organisms – are all known to predispose to severe nosocomial infections. Taken together with the stronger mortality association for infection-related tiers, these findings suggest that VAE in this population may primarily reflect a phenotype of clinically important infection that could inform future quality-improvement efforts. However, our study did not directly assess the preventability of VAE. The question of whether VAE capture potentially preventable complications rather than serving as a marker of disease severity remains debated [9, 14].

It is important to contextualize that the VAE definition was intended primarily as a surveillance tool for retrospective quality improvement and benchmarking, rather than for real-time clinical decision-making at the bedside [9, 13]. Nevertheless, our findings suggest several ways in which VAE surveillance can inform ICU practice in immunocompromised patients, particularly as the prevalence of immunosuppressive conditions and antimicrobial resistance continues to rise [22, 23]. First, our results suggest that immunocompromised status may be a risk factor for VAE development. While ventilator care bundles should be applied uniformly to all mechanically ventilated patients, recognizing immunocompromised status at ICU admission could be useful for VAE risk stratification. Second, the objective and tiered structure of the VAE framework provides a reproducible tool for benchmarking complications and evaluating outcomes in this diagnostically challenging population across different ICUs. Finally, integrating VAE surveillance with antimicrobial stewardship activities may support local audit of infection-related events, inform ICU-specific antibiograms, and help align empirical antibiotic policies with the observed PVAP pathogen and resistance profile. While detailed antibiotic exposure data around IVAC events were outside the scope of this research, future prospective studies with comprehensive antimicrobial stewardship data would be helpful to further explore the clinical importance of such surveillance.

This study has several limitations.

First, our definition of immunocompromised status, based on ICD‑10 codes at ICU admission, did not capture dynamic factors such as recent chemotherapy, cumulative corticosteroid exposure, biologic therapy, transplant recency, or current immune parameters. This may have led to nondifferential misclassification of exposure and possible underestimation of any true association between immunosuppression and VAE risk.

Second, the overall prevalence of VAE and the number of events within individual tiers were relatively low, resulting in wide confidence intervals and rendering these estimates exploratory rather than definitive. Although the association between IVAC-plus and mortality was statistically significant, it is based on only 11 events and should be interpreted with caution.

Third, attributable mortality due to VAE is difficult to ascertain. Given the high baseline mortality among immunocompromised patients without VAE, we cannot exclude the possibility that VAE serves as a marker of deterioration rather than a causal contributor to death.

Fourth, although we adjusted models for key covariates and performed a sensitivity analysis that yielded consistent estimates, we cannot exclude the possibility of residual confounding due to the observational study design. We also acknowledge that, although the proportion of excluded patients with missing MV data was low, complete-case analysis may introduce selection bias.

Finally, detailed information on specific reasons for ICU admission was not available, which limits more in‑depth group comparisons. In addition, the available follow-up constrained the ability to assess longer-term outcomes.

## Conclusion

Higher but imprecise incidence estimates did not allow us to establish a clear association between baseline immunosuppression and VAE risk. IVAC‑plus events in immunocompromised patients suggested an association with increased mortality; however, this observation should be considered exploratory given the small number of events. Larger studies with more robust exposure assessment are needed to confirm these findings.

## Supplementary Information

Supplementary Material 1.

Supplementary Material 2.

## Data Availability

The data that support the findings of this study are not openly available due to reasons of sensitivity and are available from the corresponding author upon reasonable request.

## Abbreviations

aHR: adjusted hazard ratio
aSHR: adjusted subdistribution hazard ratio
BAL: bronchoalveolar lavage
CCI: Charlson Comorbidity Index
CDC: Centers for Disease Control and Prevention
CFU: colony–forming units
CI: confidence interval
EHR: electronic health record
ETA: endotracheal aspirate
EUCAST: European Committee on Antimicrobial Susceptibility Testing
FiO₂: fraction of inspired oxygen
HAI: hospital–acquired infection
HIV: human immunodeficiency virus
ICD‑10: International Classification of Diseases, 10th Revision
ICU: intensive care unit
IQR: interquartile range
IVAC: infection–related ventilator–associated complication
LOS: length of stay
MALDI‑TOF: matrix–assisted laser desorption/ionization time–of–flight
MDR: multidrug–resistant
MMCC: Moscow Multi‑Purpose Clinical Center
MS: mass spectrometry
MV: mechanical ventilation
NHSN: National Healthcare Safety Network
PDR: pandrug–resistant
PEEP: positive end–expiratory pressure
PVAP: possible ventilator–associated pneumonia
SOFA: Sequential Organ Failure Assessment
VAE: ventilator–associated event
VA‑LRTI: ventilator–associated lower respiratory tract infection
VAP: ventilator–associated pneumonia
VAT: ventilator–associated tracheobronchitis
VAC: ventilator–associated condition
XDR: extensively drug–resistant
