# Supplementary material for: Impact of immunosuppression on the incidence of ventilator-associated events: an observational study
Source: BMC Anesthesiol. 2026 Apr 7;26:310. doi: 10.1186/s12871-026-03804-0 (PMC13185420; doi:10.1186/s12871-026-03804-0)
Supplement: Supplementary file 1 — Supplementary Material 1. [file 12871_2026_3804_MOESM1_ESM.docx]

**Supplementary file for the article: Impact of immunosuppression on the incidence of ventilator-associated events: an observational study**

**Supplementary Table 1.** ICD‑10 diagnostic codes used to define immunocompromised status and their frequencies in the study cohort.

| ICD Code | Diagnosis | N |
| --- | --- | --- |
| C34 | Malignant neoplasm of bronchus and lung | 20 |
| C16 | Malignant neoplasm of stomach | 16 |
| C18 | Malignant neoplasm of colon | 14 |
| C92 | Myeloid leukemia | 13 |
| C90 | Multiple myeloma and malignant plasma cell neoplasms | 10 |
| C25 | Malignant neoplasm of pancreas | 9 |
| C50 | Malignant neoplasm of breast | 9 |
| C95 | Leukemia of unspecified cell type | 8 |
| C83 | Diffuse non-Hodgkin lymphoma | 7 |
| C91 | Lymphoid leukemia | 6 |
| C15 | Malignant neoplasm of esophagus | 5 |
| C61 | Malignant neoplasm of prostate | 5 |
| C24 | Malignant neoplasm of biliary tract | 4 |
| C71 | Malignant neoplasm of brain | 4 |
| C85 | Other and unspecified non-Hodgkin lymphoma | 4 |
| M06 | Other rheumatoid arthritis | 3 |
| C19 | Malignant neoplasm of rectosigmoid junction | 3 |
| C53 | Malignant neoplasm of cervix uteri | 3 |
| C67 | Malignant neoplasm of bladder | 3 |
| C20 | Malignant neoplasm of rectum | 2 |
| C22 | Malignant neoplasm of liver | 2 |
| C54 | Malignant neoplasm of corpus uteri | 2 |
| C56 | Malignant neoplasm of ovary | 2 |
| C64 | Malignant neoplasm of kidney | 2 |
| C79 | Secondary malignant neoplasm of other sites | 2 |
| C80 | Malignant neoplasm without specification of site | 2 |
| B24 | HIV disease | 2 |
| M05 | Rheumatoid arthritis with rheumatoid factor | 1 |
| C03 | Malignant neoplasm of gum | 1 |
| C13 | Malignant neoplasm of hypopharynx | 1 |
| C17 | Malignant neoplasm of small intestine | 1 |
| C23 | Malignant neoplasm of gallbladder | 1 |
| C38 | Malignant neoplasm of heart, mediastinum and pleura | 1 |
| C44 | Malignant neoplasm of skin | 1 |
| C65 | Malignant neoplasm of renal pelvis | 1 |
| C73 | Malignant neoplasm of thyroid gland | 1 |
| C75 | Malignant neoplasm of other endocrine glands | 1 |
| C76 | Malignant neoplasm, other and ill-defined sites | 1 |
| C77 | Secondary and unspecified malignant neoplasm of lymph nodes | 1 |
| C78 | Secondary malignant neoplasm of respiratory and digestive organs | 1 |
| C97 | Malignant neoplasms of independent (primary) multiple sites | 1 |
| C82 | Follicular lymphoma | 1 |
| C93 | Monocytic leukemia | 1 |
| C96 | Other and unspecified malignant neoplasms of lymphoid, hematopoietic and related tissue | 1 |
| M31 | Other necrotizing vasculopathies | 1 |
| M32 | Systemic lupus erythematosus | 1 |
| M34 | Systemic sclerosis | 1 |

Note: the same patient could simultaneously have several diagnoses

**Supplementary Table 2.**  Antimicrobial resistance of ESKAPE pathogens in PVAP events among immunocompromised patients

| **Pathogen** | **N isolates** | **Carbapenem-R** | **Colistin-S** | **XDR** |
| --- | --- | --- | --- | --- |
| Acinetobacter baumannii | 5 | 5/5 (100%) | 5/5 (100%) | 5/5 (100%) |
| Klebsiella pneumoniae | 2 | 2/2 (100%) | 2/2 (100%) | 2/2 (100%) |

Note. ESKAPE pathogens include Enterococcus faecium, Staphylococcus aureus, Klebsiella pneumoniae, Acinetobacter baumannii, Pseudomonas aeruginosa, and Enterobacter species. Enterococcus faecium is part of the ESKAPE group but is not considered an eligible PVAP pathogen in the CDC VAE surveillance definitions when isolated from respiratory tract specimens (it is listed among excluded pathogens). Data are presented as number of resistant isolates/total isolates (percentage). Carbapenem‑R, carbapenem‑resistant; Colistin‑S, susceptible to colistin; XDR, extensively drug‑resistant.

**Supplementary Table 3.**  Antimicrobial resistance of ESKAPE pathogens in PVAP events among non-immunocompromised patients

| **Pathogen** | **N isolates** | **Carbapenem-R** | **Colistin-S** | **XDR** |
| --- | --- | --- | --- | --- |
| Acinetobacter baumannii | 2 | 2/2 (100%) | 2/2 (100%) | 2/2 (100%) |
| Klebsiella pneumoniae | 1 | 1/1 (100%) | 1/1 (100%) | 1/1 (100%) |
| Pseudomonas aeruginosa | 2 | 2/2 (100%) | 2/2 (100%) | 2/2 (100%) |

Note. ESKAPE pathogens include Enterococcus faecium, Staphylococcus aureus, Klebsiella pneumoniae, Acinetobacter baumannii, Pseudomonas aeruginosa, and Enterobacter species. Enterococcus faecium is part of the ESKAPE group but is not considered an eligible PVAP pathogen in the CDC VAE surveillance definitions when isolated from respiratory tract specimens (it is listed among excluded pathogens). Data are presented as number of resistant isolates/total isolates (percentage). Carbapenem‑R, carbapenem‑resistant; Colistin‑S, susceptible to colistin; XDR, extensively drug‑resistant.

**Supplementary Table 4.** Formal tests of proportional hazards assumption

| Model | Covariate | test statistic (χ²) | df | p |
| --- | --- | --- | --- | --- |
| Fine-Gray model for VAE incidence | Immunosuppression | 0.32 | 1 | 0.57 |
|  | CCI | 0.22 | 1 | 0.64 |
| Cox model for mortality with time‑dependent VAE indicator | SOFA | 0.023 | 1 | 0.88 |
|  | CCI | 0.0002 | 1 | 0.99 |

Note. Abbreviations: VAE, ventilator‑associated event; CCI, Charlson Comorbidity Index; SOFA, Sequential Organ Failure Assessment.

**Supplementary Table 5.** Sensitivity analysis of the association between immunosuppression and risk of ventilator-associated events

|  | Primary model^1^ | | | Sensitivity model^2^ | | | Unadjusted model | | |
| --- | --- | --- | --- | --- | --- | --- | --- | --- | --- |
|  | aSHR | 95% CI  for aSHR | p-value | aSHR | 95% CI  for aSHR | p-value | SHR | 95% CI  for SHR | p-value |
| VAE, overall | 1.64 | 0.82-3.30 | 0.17 | 2.07 | 1.03-4.17 | 0.042 | 1.44 | 0.75-2.78 | 0.28 |
| VAC-only | 1.17 | 0.41-3.30 | 0.77 | 1.49 | 0.58-3.83 | 0.41 | 1.06 | 0.41-2.72 | 0.90 |
| IVAC-plus | 2.22 | 0.85-5.78 | 0.10 | 2.69 | 0.92-7.84 | 0.07 | 1.91 | 0.75-4.91 | 0.18 |

^1^adjusted for Charlson Comorbidity Index (excluding immunosuppressive components)

^2^adjusted for all covariates in primary model + reason for tracheal intubation and time in hospital before mechanical ventilation episode

Note. Abbreviations: VAE, ventilator‑associated event; VAC, ventilator‑associated condition; IVAC, infection‑related ventilator‑associated complication; aSHR, adjusted subdistribution hazard ratio; SHR, subdistribution hazard ratio; CI, confidence interval.


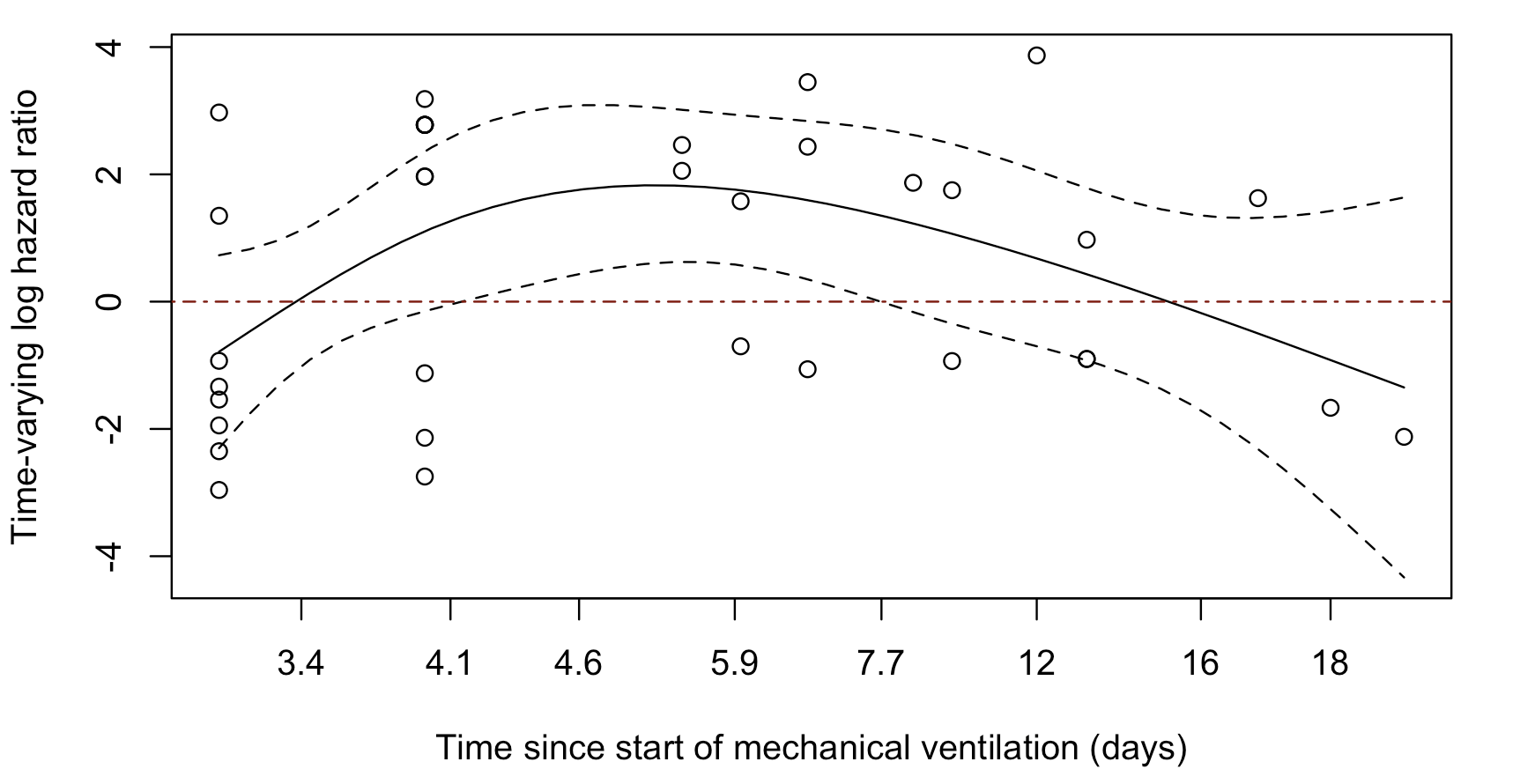


**Supplementary Figure 1.** Time‑varying coefficient plot for the Fine‑Gray model (immunosuppression covariate)

Note. Although the smoothed curve suggests some variation in the estimated log hazard ratio over time, formal tests based on scaled Schoenfeld residuals (test statistic 0.32, p = 0.57; Supplementary Table 4) did not provide statistically significant evidence of violation of the proportional subdistribution hazards assumption for immunosuppression.


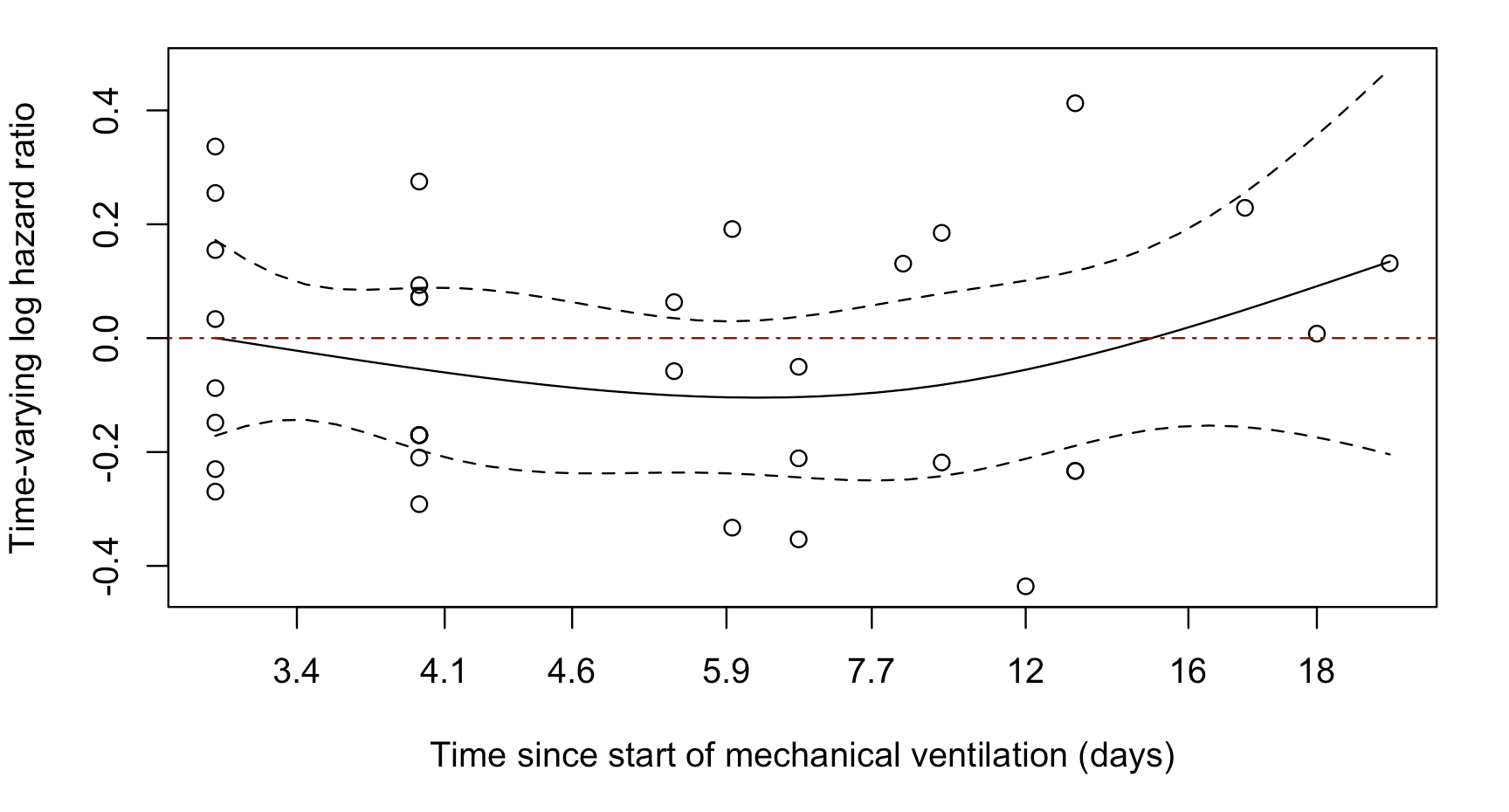


**Supplementary Figure 2.** Time‑varying coefficient plot based on scaled Schoenfeld residuals for the Fine-Gray model (Charlson Comorbidity Index covariate)

Note. All Charlson Comorbidity Index (CCI) components corresponding to immunosuppressive conditions - such as hematologic and metastatic malignancies, HIV/AIDS, and autoimmune or inflammatory diseases requiring systemic immunosuppressive therapy - are excluded from the CCI to avoid multicollinearity with the main immunocompromised exposure. The smoothed curve remains close to the horizontal zero line, indicating only minor fluctuations in the estimated log hazard ratio for the CCI over time, and formal tests based on scaled Schoenfeld residuals (test statistic 0.22, p = 0.64; Supplementary Table 4) did not provide statistically significant evidence of violation of the proportional subdistribution hazards assumption for this covariate.


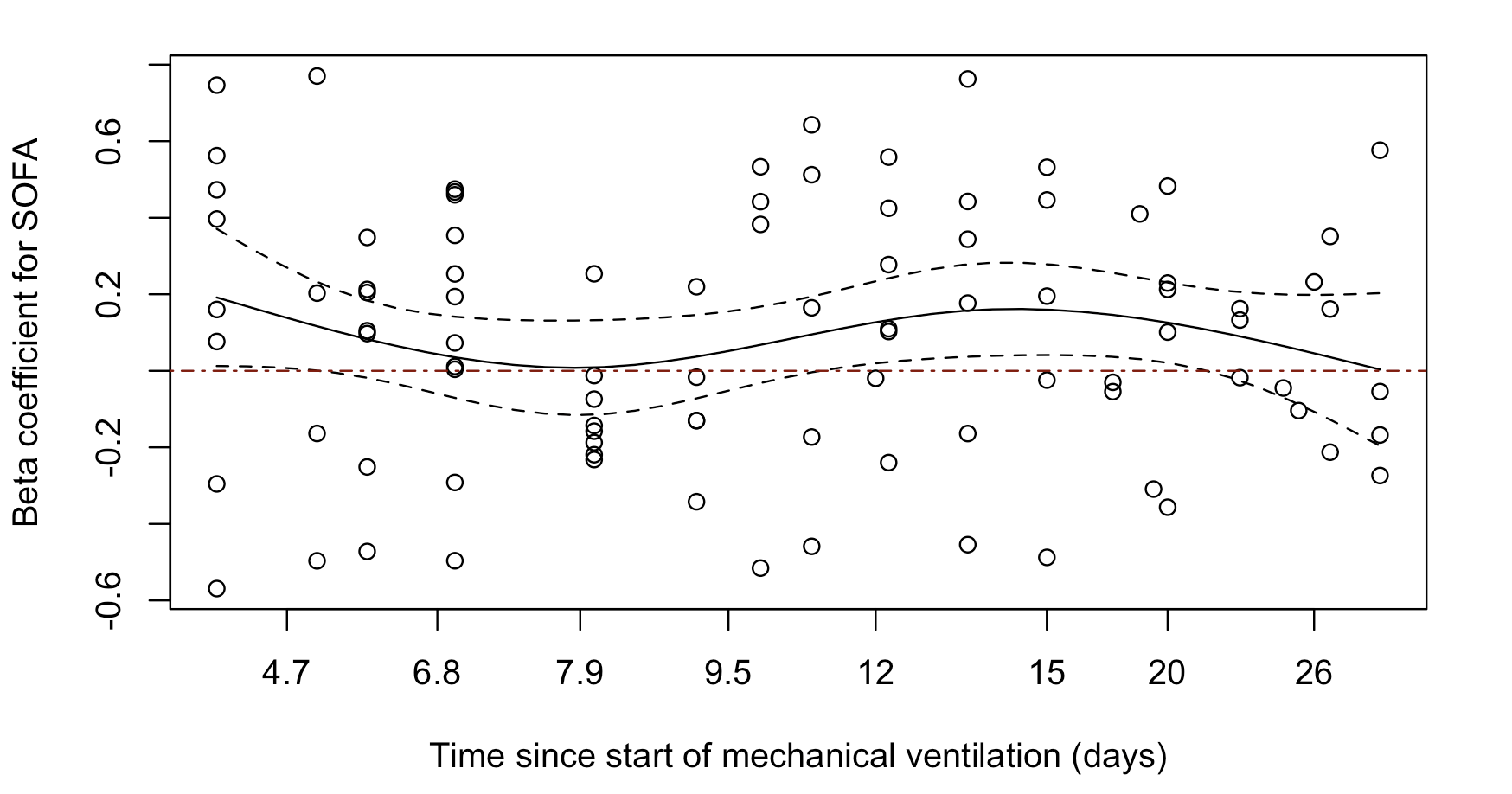
**Supplementary Figure 3.** Coefficient plot based on scaled Schoenfeld residuals for the Cox model with time‑dependent ventilator-associated events (SOFA score covariate)

Note. The smoothed curve remains close to the horizontal zero line over time, indicating no substantial time‑varying effect and no major deviation from the proportional hazards assumption for SOFA. Abbreviations: SOFA, Sequential Organ Failure Assessment.


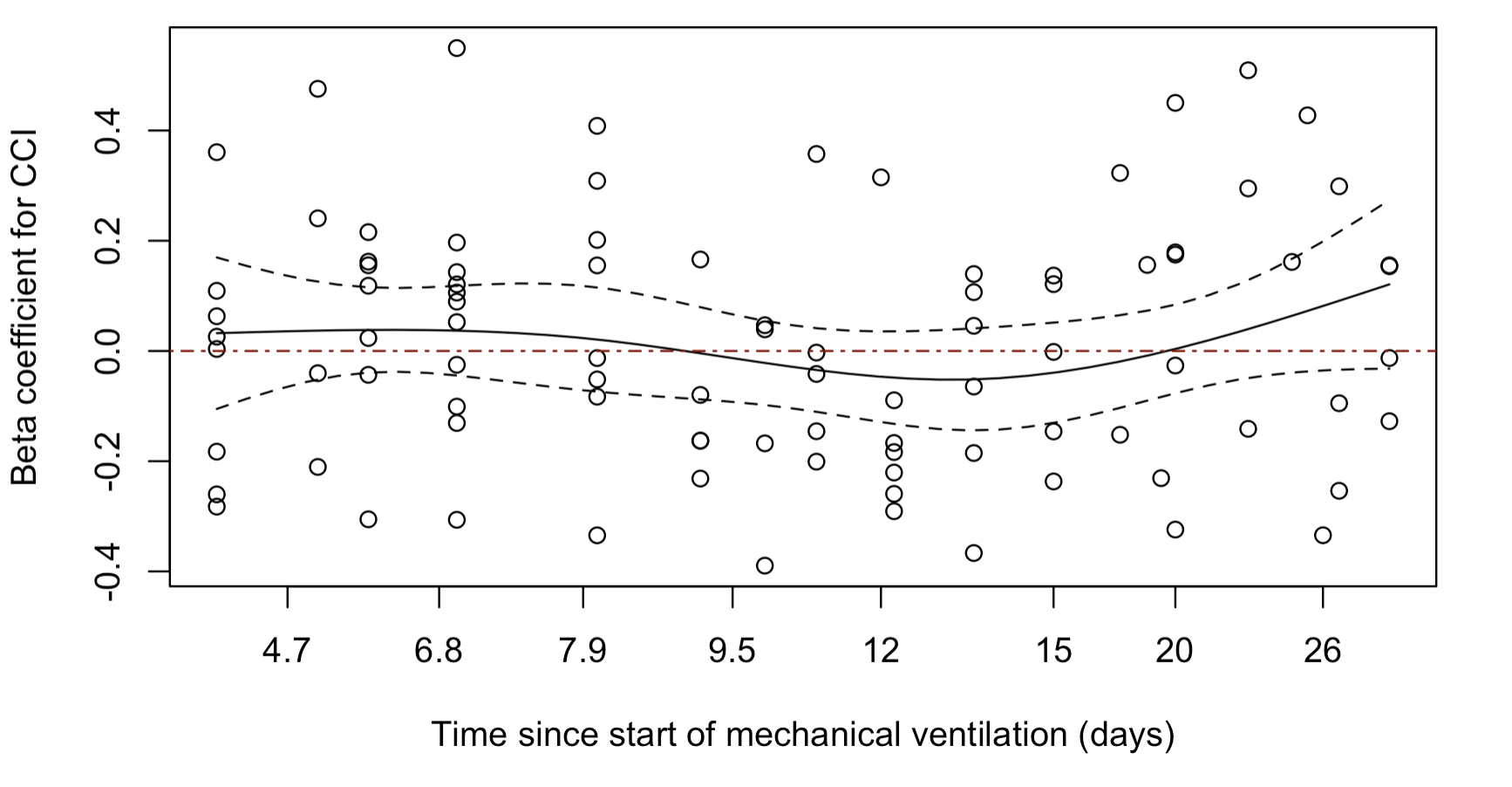
**Supplementary Figure 4.** Time‑varying coefficient plot based on scaled Schoenfeld residuals for the Cox model (CCI covariate)

Note. The smoothed curve remains close to the horizontal zero line over time, indicating only minor fluctuations in the estimated log hazard ratio for the CCI and no major deviation from the proportional hazards assumption for this covariate. Abbreviations: CCI, Charlson Comorbidity Index.
