# Supplementary material for: Impact of immunosuppression on the incidence of ventilator-associated events: an observational study
Source: BMC Anesthesiol. 2026 Apr 7;26:310. doi: 10.1186/s12871-026-03804-0 (PMC13185420; doi:10.1186/s12871-026-03804-0)
Supplement: Supplementary file 2 — Supplementary Material 2. [file 12871_2026_3804_MOESM2_ESM.pdf]

STROBE checklist for *"Impact of immunosuppression on the incidence of ventilator-associated events: an observational study"*

Item No	Recommendation	Page No	Relevant text from manuscript
**Title and abstract**			
1(a)	Indicate the study's design with a commonly used term in the title or the abstract	2	Abstract
1(b)	Provide in the abstract an informative and balanced summary of what was done and what was found	2	Abstract
**Introduction**			
2	Explain the scientific background and rationale for the investigation being reported	3	Introduction, paragraph 1-4
3	State specific objectives, including any prespecified hypotheses	3	Introduction, paragraph 5
**Methods**			
4	Present key elements of study design early in the paper	4	Methods, paragraph 1

5	Describe the setting, locations, and relevant dates, including periods of recruitment, exposure, follow-up, and data collection	4	Methods, paragraph 1
6(a)	Give the eligibility criteria, and the sources and methods of selection of participants. Describe methods of follow-up	4,5	Methods, paragraph 3, 7
6(b)	Cohort study—For matched studies, give matching criteria and number of exposed and unexposed	N/A	Not a matched study
7	Clearly define all outcomes, exposures, predictors, potential confounders, and effect modifiers	4-6	Methods, paragraph 2-5,8,9
8	For each variable of interest, give sources of data and details of methods of assessment	4,6	Methods, paragraph 1-3,6
9	Describe any efforts to address potential sources of bias	Methods, Discussion	Methods, paragraph 3,8,9

10	Explain how the study size was arrived at	NA	Sample size based on available cohort
11	Explain how quantitative variables were handled in the analyses	5	Methods, paragraph 7
12(a)	Describe all statistical methods, including those used to control for confounding	6	Methods, paragraph 8,9
12(b)	Describe any methods used to examine subgroups and interactions	6	Methods, paragraph 8,9
12(c)	Explain how missing data were addressed	5	Methods, paragraph 7
12(d)	If applicable, explain how loss to follow-up was addressed	5	Methods, paragraph 6

12(e)	Describe any sensitivity analyses	6	Methods, paragraph 8
**Results**			
13(a)	Report numbers of individuals at each stage of study	8	Results, paragraph 1
13(b)	Give reasons for non-participation at each stage	24	Figure 1
13(c)	Consider use of a flow diagram	24	Figure 1
14(a)	Give characteristics of study participants and information on exposures and potential confounders	21	Table 1
14(b)	Indicate number of participants with missing data for each variable of interest	NA	Complete-case analysis
14(c)	Summarise follow-up time	8	Results, paragraph 1
15	Report numbers of outcome events or summary measures over time	8,23	Results, paragraph 2; Table 2

16(a)	Give unadjusted estimates and, if applicable, confounder-adjusted estimates and their precision. Make clear which confounders were adjusted for and why they were included	8, 33	Results, paragraph 2,3; Supplementary Table 5
16(b)	Report category boundaries when continuous variables were categorized	N/A	Continuous variables not categorized
16(c)	If relevant, consider translating estimates of relative risk into absolute risk	N/A	N/A
17	Report other analyses done - eg analyses of subgroups and interactions, and sensitivity analyses	9	Results, paragraph 6
**Discussion**			
18	Summarise key results with reference to study objectives	10	Discussion, paragraph 1
19	Discuss limitations of the study, taking into account sources of potential bias or imprecision	12,13	Discussion, paragraph 6-9

20	Give a cautious overall interpretation of results considering objectives, limitations, multiplicity of analyses, results from similar studies, and other relevant evidence	11-13	Entire Discussion section
21	Discuss the generalisability (external validity) of the study results	14	Conclusion — "Larger multicenter prospective studies are needed to confirm these findings" (implicitly acknowledging limited generalisability)
**Other information**			
22	Give the source of funding and the role of the funders	17	N/A
